# Advances in 2D Photodetectors: Materials, Mechanisms, and Applications

**DOI:** 10.3390/mi16070776

**Published:** 2025-06-30

**Authors:** Ambali Alade Odebowale, Andergachew Mekonnen Berhe, Dinelka Somaweera, Han Wang, Wen Lei, Andrey E. Miroshnichenko, Haroldo T. Hattori

**Affiliations:** 1 Electrical Engineering, School of Engineering and Technology, The University of New South Wales, Canberra 2610, Australia; a.odebowale@adfa.edu.au (A.A.O.); a.berhe@unsw.edu.au (A.M.B.); d.liyadde_gedara@unsw.edu.au (D.S.); andrey.miroshnichenko@unsw.edu.au (A.E.M.); 2 Electrical, Electronic and Computer Engineering, The University of Western Australia, Perth 6009, Australia; han.wang@research.uwa.edu.au (H.W.); wen.lei@uwa.edu.au (W.L.)

**Keywords:** 2D material, absorption, photodetection, responsivity, surface plasmon

## Abstract

Two-dimensional (2D) materials have revolutionized the field of optoelectronics by offering exceptional properties such as atomically thin structures, high carrier mobility, tunable bandgaps, and strong light–matter interactions. These attributes make them ideal candidates for next-generation photodetectors operating across a broad spectral range—from ultraviolet to mid-infrared. This review comprehensively examines the recent progress in 2D material-based photodetectors, highlighting key material classes including graphene, transition metal dichalcogenides (TMDCs), black phosphorus (BP), MXenes, chalcogenides, and carbides. We explore their photodetection mechanisms—such as photovoltaic, photoconductive, photothermoelectric, bolometric, and plasmon-enhanced effects—and discuss their impact on critical performance metrics like responsivity, detectivity, and response time. Emphasis is placed on material integration strategies, heterostructure engineering, and plasmonic enhancements that have enabled improved sensitivity and spectral tunability. The review also addresses the remaining challenges related to environmental stability, scalability, and device architecture. Finally, we outline future directions for the development of high-performance, broadband, and flexible 2D photodetectors for diverse applications in sensing, imaging, and communication technologies.

## 1. Introduction

Photodetectors (PDs) play a critical role in modern optoelectronic systems by converting incident electromagnetic radiation—spanning the ultraviolet (UV), visible, and infrared (IR) spectra—into electrical signals. These devices underpin a wide array of applications, including optical communication networks, environmental and industrial monitoring, chemical and biological sensing, imaging systems, and medical diagnostics. Traditionally, photodetectors have been fabricated using bulk semiconductors such as silicon (Si), indium gallium arsenide (InGaAs), and mercury cadmium telluride (HgCdTe), due to their well-characterized optoelectronic properties and compatibility with mature fabrication technologies [1,2,3,4,5,6,7]. However, these conventional materials are inherently constrained by several intrinsic limitations—most notably, fixed and relatively narrow bandgaps, limited doping tunability, and high susceptibility to thermal noise. These limitations collectively hinder the performance of conventional photodetectors in terms of responsivity, spectral tunability, and detection efficiency, especially under low-light or ambient operating conditions.

The emergence of two-dimensional (2D) materials has opened new frontiers in photodetector design and performance. Owing to their atomically thin structure and quantum confinement effects, 2D materials offer extraordinary physical and chemical properties that are highly advantageous for optoelectronic applications [8,9]. These include broadband optical absorption, ultrafast carrier dynamics, high carrier mobility, mechanical flexibility, and facile integration with diverse substrates. Notable examples of 2D materials with promising photodetection capabilities include graphene, transition metal dichalcogenides (TMDCs), black phosphorus (BP), hexagonal boron nitride (h-BN), and emerging III–V monolayers. Furthermore, vertical and lateral van der Waals (vdW) heterostructures—formed by stacking or patterning different 2D materials—enable novel functionalities that are unattainable in traditional bulk systems [10,11,12,13,14]. A particularly attractive feature of 2D materials is their compatibility with silicon-based platforms. Unlike bulk III–V semiconductors, which suffer from lattice and thermal expansion mismatches when integrated with silicon substrates, 2D materials can be transferred onto a variety of surfaces using vdW assembly techniques, eliminating the need for lattice matching [15,16]. This integration strategy offers a scalable pathway for the realization of high-performance, CMOS-compatible photonic systems, particularly for mid-infrared (MIR) and terahertz (THz) applications where conventional materials fall short [10].

This review provides a comprehensive overview of different 2D photodetectors and their various detection mechanisms, with a focus on their underlying physical principles and relevance to optoelectronic device performance. Specifically, we discuss key mechanisms including the photovoltaic effect, photothermoelectric effect, photoconductive response, bolometric effect, carrier generation and trapping, built-in electric fields in heterostructures, and plasmon-enhanced detection. Each mechanism is examined in the context of specific material properties and device architectures. By elucidating these photodetection pathways, this review aims to highlight the current advancements and future potential of 2D materials in enabling next-generation photodetectors for diverse spectral regimes and application domains.

## 2. Mechanisms of Photodetection in 2D Materials

Photodetection in 2D materials relies on the conversion of incident photons into measurable electrical signals, a process that can proceed through various physical mechanisms. The diversity of these mechanisms reflects the rich optoelectronic properties of 2D materials, which stem from their atomically thin geometry, tunable band structures, and enhanced light manipulations. Understanding these mechanisms is crucial for tailoring photodetector performance across specific application domains such as broadband detection, ultrafast response, or high sensitivity. This section provides a detailed discussion of these mechanisms, highlighting their underlying physics and potential applications [17].

**Figure 1:**
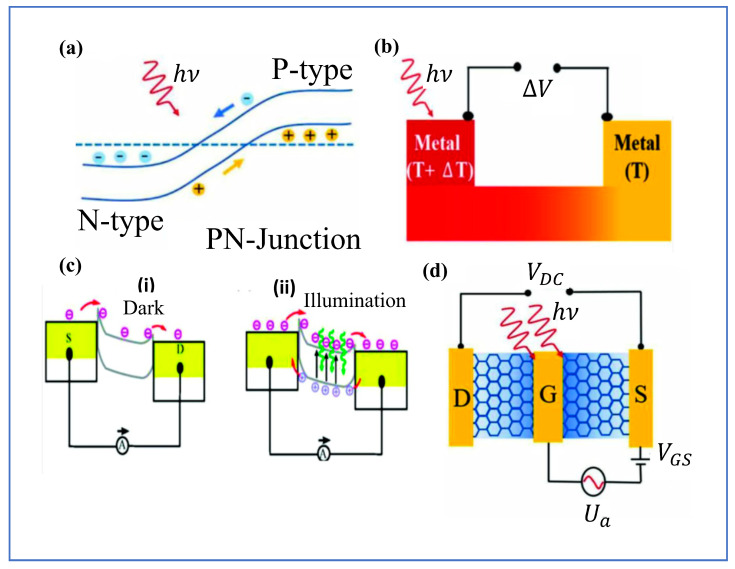
Illustration of various photodetection mechanisms in 2D materials: (**a**) photovoltaic effect, where built−in electric fields separate photogenerated carriers; (**b**) photothermoelectric effect, where a temperature gradient induces a voltage via the Seebeck effect; (**c**) Illustration of the photoconductive effect: (i) Dark condition: In the absence of light, very few charge carriers are available, resulting in low or negligible current flow between the source (S) and drain (D) (ii) Under illumination: Incident light generates additional electron−hole pairs (green arrows), increasing the carrier concentration and enhancing the conductivity of the material, leading to a measurable photocurrent. and (**d**) bolometric effect, where light−induced heating changes the material’s resistance [19].

A fundamental mechanism is the photovoltaic effect, which occurs when photogenerated electron–hole pairs are separated by an internal electric field, typically present at p–n junctions or heterostructure interfaces [18]. Alternatively, an external electric field, induced by applying a bias voltage between the source and drain, can also drive this effect. However, this approach is less desirable as it introduces dark current, as illustrated in Figure 1a [19]. This built-in field, arising from work function differences or local chemical doping, drives charge carriers in opposite directions, generating a photocurrent without the need for an external bias [20]. In particular, the direction of current depends on the type of junction (pn vs. np), and the process benefits from carrier multiplication effects that improve quantum efficiency [21].

Closely related in outcome but different in principle is the photothermoelectric effect, which exploits spatial variations in light absorption to create temperature gradients [22]. These gradients induce a voltage through the Seebeck effect. Here, the difference in thermoelectric coefficients between different regions of the device—often engineered via asymmetric contacts or spatially varying materials—leads to a measurable photovoltage (see Figure 1b [19]). Unlike the photovoltaic effect, which is driven by electric fields, the photothermoelectric effect leverages thermal energy, making it particularly relevant in materials with high thermopower. According to the Seebeck effect, this temperature difference alters the electronic properties of the material, leading to a measurable potential difference [23]. The induced photothermoelectric voltage, expressed as $V=(S_{2}-S_{1})\Delta T$, depends on the thermoelectric coefficients of the material ($S_{1}$ and $S_{2}$) and the temperature gradient ΔT across different regions. The magnitude of these coefficients is closely related to the conductance of the material [23].

Another widely utilized mechanism is the photoconductive effect, which occurs when the absorption of light enhances the electrical conductivity of a semiconductor. Incident photons excite electrons from the valence band to the conduction band, generating electron–hole pairs and increasing the charge carrier density (see Figure 1c [8]. Under an applied electric field, these free carriers contribute to a measurable photocurrent. Unlike photovoltaic devices, which operate at zero bias, photoconductive devices require an external electric field to facilitate charge transport and photocurrent generation [20]. Additionally, carrier recombination may occur, affecting the efficiency of the process.

In contrast to the carrier-driven mechanisms above, the bolometric effect relies on the temperature-dependent resistivity of the material. This occurs when there is variation in electrical resistance of a material due to temperature changes induced by incident electromagnetic radiation. This effect is leveraged in bolometric photodetectors, where absorbed light energy is converted into an electrical signal [24]. As shown in Figure 1d, when light is absorbed, the resulting temperature increase alters the resistivity of the material, leading to a photocurrent that scales with the applied voltage [19]. This resistivity change occurs through two primary mechanisms: (1) a temperature-dependent variation in carrier mobility and (2) a change in carrier contribution to the current. The sensitivity and response time of bolometric devices are primarily influenced by the material’s thermal resistance ($R_{Th}$) and heat capacity ($C_{h}$) [24].

In many 2D materials, carrier generation and trapping effects also play a key role. Upon light absorption, electron–hole pairs are generated. However, structural defects or impurity states can trap one type of carrier (typically electrons or holes), prolonging the lifetime of the opposite carrier and amplifying the photocurrent. This leads to photogating behavior, where the trapped carriers modulate the conductivity of the channel over longer timescales, resulting in high responsivity albeit with slower response times. The formation of heterostructures between different 2D materials introduces additional photodetection pathways. Due to band alignment and differences in work functions, built-in electric fields form at the interfaces, enhancing charge separation and reducing recombination. These internal fields improve collection efficiency and reduce power consumption, making heterostructure-based photodetectors especially attractive for integrated optoelectronics [25].

To further enhance light absorption in atomically thin materials, plasmonic nanostructures—metallic components that support surface plasmon resonances—can be integrated with 2D layers. Localized surface plasmons (LSPs) and surface plasmon polaritons (SPPs) are collective oscillations of electrons on the surface of metallic nanostructures excited by light. These oscillations amplify the local electromagnetic field, boosting light–matter interactions and enhancing the performance of 2D photodetectors. As a result, integrating plasmonic nanostructures with 2D materials can concentrate light into sub-wavelength volumes, enhancing local electromagnetic fields and thereby increasing light absorption and photocurrent generation in photodetectors. Hence, 2D materials such as graphene and transition metal dichalcogine (TMDC) monolayers exhibit low light absorption due to their atomically thin structure, limiting their efficiency in converting light into electrical signals. As a result, hybridizing these materials with plasmonic nanostrucutres is essential for enhancing the perfomance of photodetectors. For example, Yuan Liu et al. demonstrated the feasibility of pristine graphene as compared with integration with gold nanostrucures. Although pristine graphene photodetectors are ultrafast and broadband, they suffer very low quantum efficiency (0.1–0.2%). By integrating plasmonic gold nanostrucutres onto graphene, a drastic photocurrent enhancement of up to 1500% was achieved, enabling multicolor photodetection [26].

To better understand the practical implications and performance of each photodetection mechanism in 2D materials, Table 1 presents a comparative summary highlighting their operational principles, bias requirements, key advantages, limitations, and typical applications. This comparison provides a clear overview of the strengths and trade-offs associated with each mechanism.

## 3. 2D Materials for Photodetection

In recent years, the exploration of 2D materials has led to significant breakthroughs in the development of high-performance photodetectors. Due to their atomically thin nature, tunable bandgaps, and enhanced light–matter interaction, 2D materials offer a versatile platform for designing photodetectors across a broad spectral range: from ultraviolet to far infrared. This section provides a comprehensive overview of various classes of 2D materials that have demonstrated strong potential for photodetection applications.

### 3.1. Graphene-Based Photodetectors

Graphene-based photodetectors have emerged as a promising class of optoelectronic devices due to graphene’s exceptional electrical, thermal, and optical properties. In the mid-infrared (MIR) regime, where many conventional semiconductors face limitations, graphene offers broadband absorption, ultrafast carrier dynamics, and compatibility with silicon-based platforms—making it particularly attractive for integrated photonic applications such as on-chip sensing and spectroscopy. Unlike other 2D materials, such as black phosphorus (BP), which suffer from environmental instability and fabrication challenges, graphene’s chemical stability and zero bandgap nature enable detection across a wide spectral range, from the far-infrared to the ultraviolet. This section highlights recent advancements in waveguide-integrated graphene photodetectors, with a focus on device configurations, photoresponse mechanisms, and performance metrics that underscore graphene’s suitability for MIR applications.

In the MIR region, graphene has been successfully integrated into the Si substrate which has demonstrated superior performance compared with 2-D black phosphorus (BP) [11], which suffers from surface instability due to chemical degradation [12], limiting its practicality for on-chip sensing applications. Graphene is a gapless semiconductor that is capable of detecting electromagnetic radiation from the far infrared to the ultraviolet [27]. Researchers have reported on-chip MIR graphene photodetectors [13,28,29,30,31,32,33]. Wang et al. reported the development of a graphene/silicon heterojunction photodetector by integrating a graphene layer onto a silicon optical waveguide fabricated on a silicon-on-insulator (SOI) platform. This configuration enabled the efficient absorption of evanescent light traveling parallel to the graphene surface, allowing the device to operate effectively across the near- to mid-infrared spectrum. At a bias voltage of 1.5 V, the photodiode demonstrated a responsivity of 0.13 A/W for incident light with a wavelength of 2.75 μm at room temperature [29]. In a related work, Cheng et al. fabricated heterostructure waveguide photodetectors by transferring chemical vapor deposition (CVD)-grown graphene onto silicon-on-sapphire (SOS) waveguides. These devices exhibited responsivities of 0.9 mA/W at 1.55 μm and 4.5 mA/W at 2.75 μm, highlighting the potential of graphene-based integration for broadband infrared photodetection [28].

**Figure 2:**
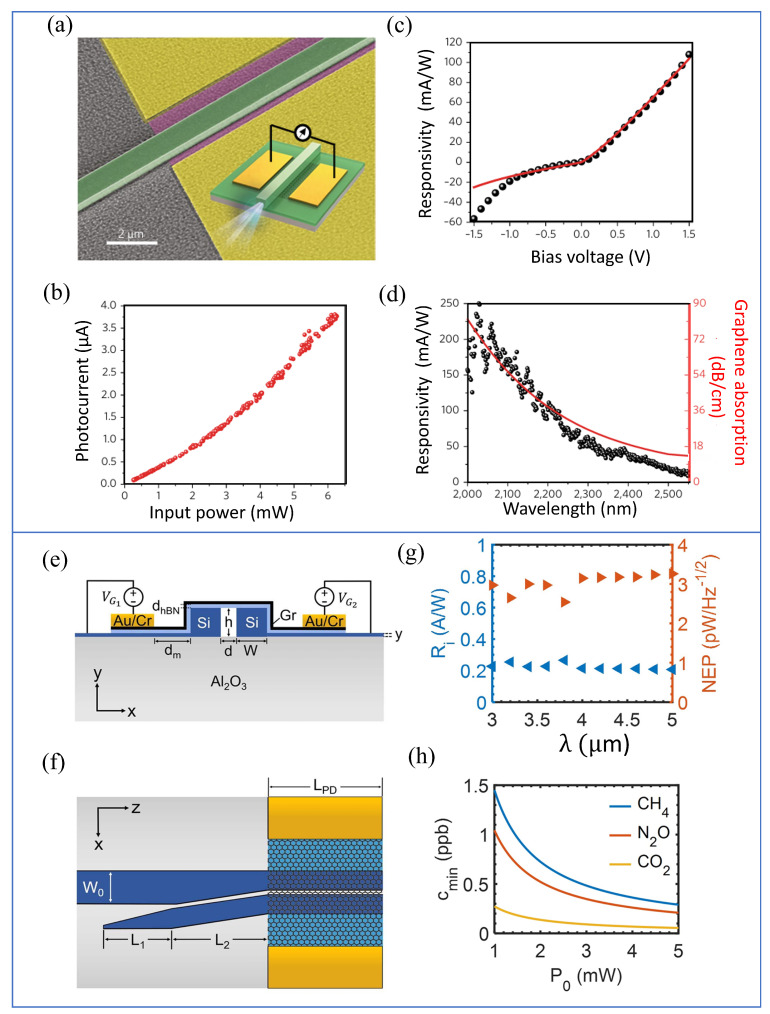
Waveguide −integrated graphene mid−infrared (MIR) photodetectors and their performance metrics. (**a**) Scanning electron microscope (SEM) tilted−view micrograph of a waveguide-integrated graphene photodetector, with the inset showing a schematic illustration of the device structure comprising a chalcogenide glass waveguide patterned atop monolayer graphene. (**b**) Zero−bias photocurrent measured as a function of incident optical power at a wavelength of 2185 nm, demonstrating the device’s light detection capability without external bias. (**c**) Responsivity of the detector device to 2185 nm waveguide input. (**d**) Mid−infrared broadband spectral dependences of the detector’s responsivity (at 1.5 V bias) and calculated optical absorption in the graphene layer. (**e**,**f**) On−chip graphene photodetector: (**e**) front view; (**f**) top view. (**g**) Current responsivity ($R_{i}$, left axis) and noise equivalent power (NEP, right axis) as functions of wavelength (λ) in the mid−infrared range. (**h**) Minimum detectable concentration ($c_{min}$) as a function of the source power ($P_{0}$) [9,33].

Lin et al. presented an efficient approach for fabricating graphene-based photodetectors by directly depositing and patterning chalcogenide glass (ChG) waveguides and metal electrodes onto monolayer graphene synthesized via chemical vapor deposition (CVD), thereby simplifying the integration of the photodetector with the optical waveguide system (Figure 2a, inset) [33]. To assess the device’s performance, mid-infrared light with transverse electric (TE) polarization was launched into the waveguide, and the generated photocurrent was recorded under zero-bias conditions (Figure 2b). Graphene’s photoresponse typically arises from a combination of three fundamental mechanisms: the photothermoelectric effect, the photovoltaic (PV) effect, and the bolometric effect. To determine which of these played the dominant role in the observed photocurrent, the authors compared experimental results against theoretical models for each mechanism. Among the three, the photovoltaic effect model exhibited the best agreement with the measured data (Figure 2c). From the fitting, a hot carrier relaxation time of 2.3 picoseconds was extracted, which is consistent with values reported in earlier studies [34,35]. Additionally, the responsivity of the device was found to increase with applied bias voltage, attributed to improved efficiency in charge carrier collection. An asymmetry in the photocurrent response curve was observed, which the authors linked to the lateral displacement of the waveguide relative to the graphene channel (Figure 2a). Deviations from the photovoltaic model at negative biases exceeding −1 V were likely a result of the intrinsic disparity in electron and hole mobilities in graphene [36]. The photodetector exhibited broadband sensitivity throughout the entire tuning range of the laser source (2.0–2.55 μm), achieving a peak responsivity of 250 mA/W at a wavelength of 2.03 μm (Figure 2d). This level of performance is competitive with, and in some instances surpasses, other state-of-the-art waveguide-integrated graphene photodetectors developed for both mid-infrared [29] and near-infrared applications [37,38]. Notably, achieving responsivities above 10 mA/W in the mid-infrared (MIR) range remains a significant challenge for graphene-based detectors.

In a related advancement, Mohammed Alaloul and colleagues proposed a mid-wave infrared (MWIR) graphene photodetector integrated with silicon-on-sapphire slot waveguides, optimized for on-chip gas sensing through absorption spectroscopy (Figure 2e,f). The device capitalizes on the strong interaction between the optical mode and analyte gases in the slotted air region of the waveguide, which is crucial for high-sensitivity detection. The authors evaluated the device by analyzing its current responsivity (R_*i*_) and detection capabilities for trace gases such as carbon dioxide (CO_2_), methane (CH_4_), and nitrous oxide (N_2_O). As shown in Figure 2g, the R_*i*_ across the MWIR band remained relatively uniform, ranging between 0.21 and 0.26 A/W. Minor fluctuations in the R_*i*_ were attributed to variations in coupling efficiency and wavelength-dependent absorption by graphene in the waveguide-integrated setup. The obtained responsivity is much greater than previously reported MIR graphene-based photodetectors [29,37,38], which is critical to enhance the sensitivity of the device in detecting low concentrations of target gases. Impressively, the system demonstrated the ability to detect minimum concentrations ($c_{min}$) as low as 0.14 parts per billion (ppb) for CO_2_, 0.52 ppb for N_2_O, and 0.72 ppb for CH_4_ under a source power of 2 mW (Figure 2h) [9].

Compared with other 2D material-based photodetectors, such as those leveraging BP or TMDs, graphene-based devices offer a compelling balance of broadband sensitivity, fast response time, and CMOS compatibility. While BP devices may exhibit higher intrinsic responsivity due to their finite bandgap, they are often hindered by rapid degradation under ambient conditions, making long-term operation challenging. In contrast, graphene devices—particularly those integrated with silicon and chalcogenide waveguides—have demonstrated reliable operation in the MIR range with responsivities exceeding 250 mA/W and detection limits down to sub-ppb levels for trace gases. These results not only match but in some cases surpass the performance of other 2D photodetectors, reinforcing graphene’s position as a robust and scalable material for next−generation infrared sensing technologies.

### 3.2. Transition Metal Dichalcogenides Photodetectors

**Figure 3:**
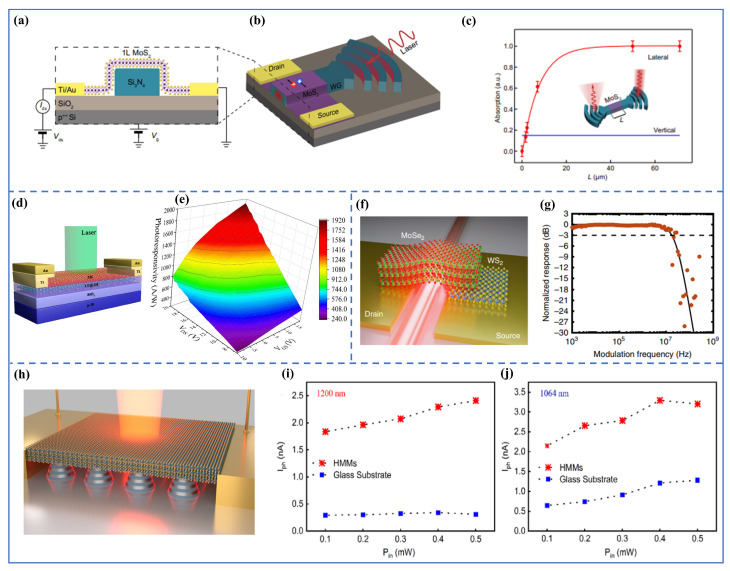
TMDC photodetectors. (**a**) Schematic of a MoS_2_-based photodetector integrated into a photonic circuit, where monolayer MoS_2_ is directly exfoliated onto the waveguide. (**b**) Illustration of light coupling achieved by focusing a 647 nm continuous-wave (CW) laser onto the diffraction grating. The light propagates through the Si_3_N_4_ waveguide and generates electron–hole pairs in MoS_2_, leading to photocurrent generation in the biased monolayer. (**c**) Influence of the MoS_2_ monolayer and metal contacts on waveguide light propagation. Experimental data compare lateral and vertical absorption as a function of flake length [41]. (**d**) Schematic showing gate and source–drain bias modulation of the photodetection performance in a vertically stacked 2H-MoS_2_/1T@2H-MoS_2_ device. (**e**) A 3D view of the photoresponsivity mapping of this device under varying gate and source–drain biases, measured at an illumination power density of 2.35 mW/cm^2^ [42]. (**f**) Schematic of a MoSe_2_–WS_2_ heterostructure photodetector transferred onto a silicon nitride waveguide. (**g**) Frequency response of the photodetector with modulation frequency of incident light is swept, revealing a −3 dB cutoff at ∼20 MHz. The solid line fits an RC low-pass filter model [43]. (**h**) Schematic of a hyperbolic metamaterial (HMM) nanocavity integrated with a 2D TMDC layer. (**i**,**j**) Photocurrent as a function of incident power at resonance (1200 nm) and off-resonance (1064 nm), respectively. A ∼7.5-fold enhancement is observed at ∼0.3 mW for on-resonance excitation, while the off-resonance case shows ∼2.5-fold enhancement at similar power levels [44].

TMDC monolayers such as molybdenum disulfide (MoS_2_), tungsten disulfide (WS_2_), tungsten diselenide (WSe_2_), molybdenum diselenide (MoSe_2_), and molybdenum ditelluride (MoTe_2_) exhibit direct bandgap semiconductor characteristics in the visible to near-infrared spectrum when reduced to a single atomic layer [39,40]. Their intrinsic properties, including high exciton binding energies (>300 meV) and mechanical flexibility, have enabled device innovations with extraordinary responsivity, detectivity, and tunability. When these TMDCs are operated in photovoltaic mode, a built-in electric field at a junction (e.g., TMDC *p*–*n* junction or Schottky barrier) separates electrons and holes, generating photocurrent even under very low bias. Such TMDC photodiodes typically offer faster, low-noise operation due to suppressed dark current, though they generally exhibit lower gain compared with phototransistors. Figure 3a,b illustrate a representative device structure where monolayer MoS_2_ is integrated on a silicon nitride waveguide: under lateral evanescent illumination, electron–hole pairs generated in the MoS_2_ are collected by biasing the channel, thereby converting guided light into electrical signals [41]. As a result, coupling light laterally via a waveguide significantly enhances absorption in the atomically thin monolayer compared with normal incidence, effectively approaching unity absorption for sufficiently long interaction lengths (Figure 3c). The versatility of TMDCs is also evident in configurations ranging from monolayers to vertically stacked heterostructures. For instance, Wang et al. demonstrated a vertically stacked 2H-MoS_2_/1T@2H-MoS_2_ heterojunction that achieves a responsivity of 1917 A/W and detectivity on the order of 10^11^ Jones through phase-engineered internal junctions and bias modulation [42]. As shown in Figure 3d–e, the 1T metallic phase in the heterostructure facilitates efficient carrier transport, while the 2H semiconducting phase enables light absorption, demonstrating how phase engineering can optimize performance and achieve high responsivity. On the other hand, forming van der Waals heterojunctions is a key strategy to achieve both high responsivity and fast response. For instance, Gherabli et al. demonstrated a lateral *p*–*n* heterojunction between *p*-type MoSe_2_ and *n*-type WS_2_ monolayers integrated on a silicon nitride (SiN) waveguide, resulting in a self-driven photodiode with ultra-low dark current (∼50 pA) and a peak responsivity of approximately 0.9 A/W at 780 nm [43]. As shown in Figure 3f–g, the MoSe_2_–WS_2_ photodetector exhibits strong optical field confinement when coupled with the waveguide, significantly enhancing light absorption within the TMDC region. Consequently, as illustrated in Figure 3g, the device demonstrates a temporal photoresponse with a −3 dB bandwidth of ∼20 MHz and sub-30 ns rise and fall times, confirming its potential for high-speed on-chip applications.

Other TMDCs, such as WSe_2_, MoSe_2_, and MoTe_2_, have also enabled diverse device architectures and photonic enhancements. For instance, hybridizing a MoTe_2_/MoS_2_ bilayer with metamaterial (HMM) nanocavities led to an 8-fold increase in photocurrent (see Figure 3h–j). This enhancement is attributed to the localized electric fields around each nanocavity, which leads to enhanced absorption in the 2D materials [44]. Doping strategies have also yielded transformative performance gains. Kang et al. investigated the impact of self-assembled monolayer (SAM) doping on the performance of WSe_2_- and MoS_2_-based photodetectors. By employing octadecyltrichlorosilane (OTS) for *p*-doping WSe_2_ and aminopropyltriethoxysilane (APTES) for *n*-doping MoS_2_, they achieved significant enhancements in device performance. Specifically, the responsivity of WSe_2_ photodetectors increased from 517.2 A/W to $1.45\times 10^{4}$ A/W, and that of MoS_2_ devices improved from 219 A/W to $5.75\times 10^{3}$ A/W, representing approximately 28-fold and 26-fold enhancements, respectively [45]. These improvements were attributed to the non-destructive nature of SAM doping, which effectively modulates the carrier concentration without impairing carrier transport properties.

Furthermore, TMDCs offer several key advantages over other 2D material-based photodetectors. For example, TMDC photodetectors are crucial due to their tunable bandgap compared with graphene. Graphene suffers from its fundamental limitations due to its zero bandgap and semimetallic nature. Additionally, while graphene photodetectors are known for their ultrabroadband spectral coverage and ultrafast response, they typically exhibit high dark current and low intrinsic responsivity owing to minimal optical absorption [8]. In contrast, TMDC monolayers possess sizable and tunable direct bandgaps in the visible to near-infrared (NIR) range, along with strong excitonic absorption, leading to significantly lower off-state currents and inherently higher photoresponsivity. Graphene’s light absorption, approximately 2.3% per layer, is notably lower than that of TMDCs, which can exhibit an order of magnitude greater absorption at excitonic resonances [8]. As a result, TMDC-based photodetectors often achieve superior signal-to-noise ratios. For instance, monolayer MoS_2_ phototransistors can reach responsivities at the A/W level without requiring optical cavities, which is higher than graphene photodetectors. Additionally, TMDC-based photodetectors are also more stable than their black phosphorus (BP) counterparts. Despite BP offering a tunable mid-IR bandgap and high mobility, it is chemically unstable under ambient conditions [46].

The unique combination of high sensitivity and integrability makes TMDC photodetectors attractive for a broad range of technological applications. For instance, two recent studies have demonstrated the potential of MoS_2_-based active pixel sensor (APS) arrays for next-generation imaging. Dodda et al. developed a monolayer MoS_2_ phototransistor array that integrates image capture and in-sensor processing within a compact, low-power platform (<1 pJ/pixel), achieving ultra-high responsivity (∼3.6 × 10^7^ A/W), detectivity (∼5.6 × 10^13^ Jones), and dynamic range (∼80 dB) using gate-tunable persistent photoconductivity. Their approach eliminates the need for peripheral circuitry for analog de-noising and reset, making it ideal for edge computing in IoT systems [47]. Additionally, Hong et al. fabricated a large-area bilayer MoS_2_ APS using a transfer-free growth method, achieving responsivity up to 119 A/W and demonstrating high pixel uniformity and signal-to-noise ratio [48]. They attributed this enhancement to photogating via hole trapping at subgap states, confirmed through simulation and spectroscopic analysis. Together, these works highlight the feasibility of scalable, high-performance, and energy-efficient TMDC-based imaging platforms. Due to their atomic thickness, TMDCs can be vertically stacked or tiled on focal plane arrays without adding significant bulk, facilitating multispectral imaging through the use of different TMDC materials tuned to distinct spectral bands. Moreover, TMDC photodetectors are increasingly used as in situ optical read-heads in chemical and biological sensing. For example, Park et al. developed a 4 × 4 mini-array of few-layer MoS_2_ integrated with microfluidic enzyme chambers to detect the blood biomarker D-lactate in 2.5 μL samples within 10 min, achieving sub-μg mL^−1^ sensitivity due to high responsivity and pixel uniformity [49].

Photodetectors based on thin TMDCs combine direct bandgap semiconducting behavior, strong optical absorption, and atomic-scale thickness to deliver high responsivity, low dark current, and easy integration onto rigid, flexible, or photonic platforms. Over the past decade, they have advanced from lab-scale flakes to wafer-level arrays. Monolayer MoS_2_ phototransistors now reach responsivities beyond 10^6^ A/W; WSe_2_ and MoTe_2_ *p*–*n* diodes offer low dark current and multi-gigahertz bandwidths suitable for telecom applications [47,48]. As a result, TMDC photodetectors offer many advantages, especially in terms of flexibility and system-level integration. Further progress depends on innovations in materials and device engineering. Optimized contacts such as edge-engineered metals or 2D conductors could reduce series resistance and noise. Doping strategies, whether chemical or electrostatic, can tune spectral response while preserving carrier mobility. Heterostructures combining TMDCs with black phosphorus, perovskites, quantum dots, or plasmonic resonators offer enhanced gain, broader spectral range, and multifunctionality. In particular, integrating TMDC monolayers with ultrathin plasmonic metamaterials can induce strong local electric field enhancements [50,51], significantly increasing optical absorption and overall photodetector performance. Techniques such as strain engineering and the use of high-κ dielectrics can further improve carrier dynamics and reduce flicker noise. Scalable, transfer-free growth on CMOS-compatible substrates remains key to industrial adoption. These strategies will enable TMDC photodetectors with high speed, sensitivity, and mechanical adaptability for future optoelectronic devices.

### 3.3. Black Phosphorus (BP) Photodetectors

Black phosphorus (BP), a layered 2D semiconductor with a tunable bandgap ranging from 0.3 eV (bulk) to ≈2 eV (monolayer), has emerged as a leading candidate for broadband photodetection, particularly in the infrared (IR) and midwave infrared (MWIR) spectral ranges [52]. Its anisotropic carrier mobility, high absorption coefficient, and out-of-plane electric field response provide unique advantages over traditional semiconductors and even other 2D materials [52]. Recent work has focused on enhancing the material’s optoelectronic response, air stability, and spectral tunability, positioning BP as a key enabler for next-generation photodetectors.

Several studies have focused on improving the synthesis and processing of BP for device fabrication. Song et al. demonstrated uniform, large-area Te_2_-regulated black arsenic phosphorus films that achieved remarkable self-powered IR detection with detectivity up to $8.5\times 10^{10}$ Jones [53]. Similarly, Wan et al. developed a chemical vapor transport method to grow wafer-scale BP, enabling memristive behavior and neuromorphic functions in optoelectronic systems [54]. These fabrication strategies are critical for transitioning BP-based photodetectors from the lab to scalable technologies. To overcome BP’s environmental instability, multiple chemical and physical passivation techniques have been reviewed. Zhang et al. comprehensively discussed encapsulation and doping strategies that significantly reduce photo-oxidation, extending device lifetimes in ambient conditions [55]. Device engineering has played a crucial role in enhancing performance metrics like responsivity, response time, and spectral coverage. Also integration with stable materials has also been proven to be effective. Zhou et al. fabricated solution-processed BP/RGO Schottky junctions that operate at temperatures up to 400 K while maintaining near-IR responsiveness up to 2200 nm [56]. Shen and Hou developed asymmetric Schottky BP transistors achieving detectivity as high as $2.47\times 10^{11}$ Jones in the 400–2200 nm range [57]. Advanced designs such as twisted bilayers (Chen et al., 2024) introduced tunable photoluminescence and the bulk photovoltaic effect, offering new pathways to manipulate carrier dynamics without external bias [58]. Heterostructure-based devices have seen significant advancements. Hu and team engineered heterogeneous Z/S scheme BP interfaces to enhance interfacial charge transfer, boosting photogenerated carrier separation efficiency [59]. Likewise, BP-MoS_2_ diodes, integrated with metasurfaces, demonstrated superior MWIR absorption in the 3–5 μm range (Lien et al., 2023) [60]. These vertical and lateral heterojunctions significantly enhance optical gain and offer spectral selectivity.

**Figure 4:**
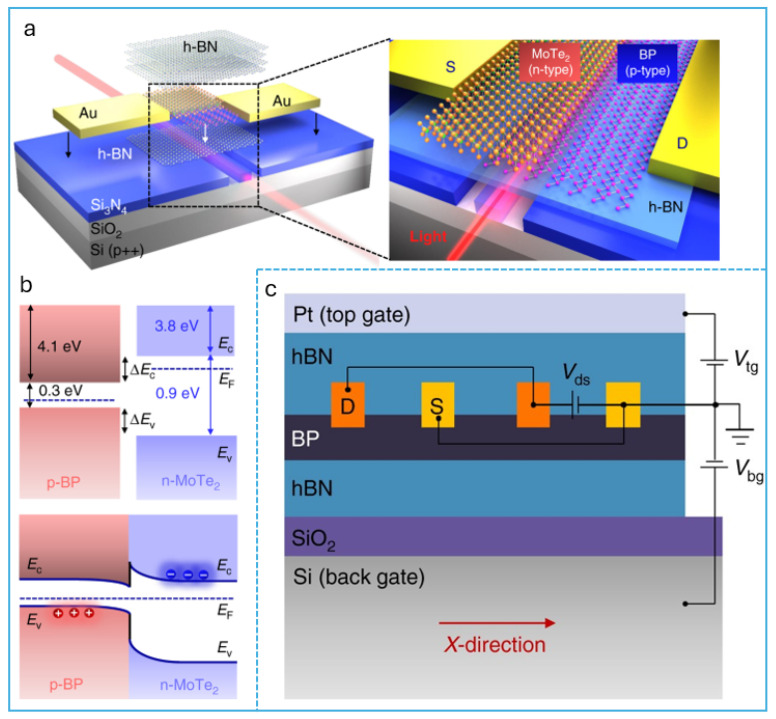
Different type of BP devices’ architecture. (**a**) Schematic of the waveguide-integrated van der Waals PN heterojunction photodetector composed of stacking p-doped BP and n-doped MoTe2, which are evanescently coupled with the guiding mode of the silicon nitride waveguide [68]. (**b**) **Top**: Band profiles of BP and MoTe2 layers in the non-equilibrium state. **Bottom**: Band alignment of BP/MoTe2 PN heterojunction in the thermal equilibrium state [68]. (**c**) Structure of the tunable BP mid-IR photodetector based on a dual-gate transistor configuration [69].

Polarization sensitivity and directionality are also intrinsic to BP’s anisotropic nature. Hao and co-workers fabricated a Dember effect-based anisotropic photodetector with polarization ratios exceeding 100, suggesting potential use in imaging and sensing applications [61]. Similarly, Li et al. explored ferroelectric black bismuth (a BP analog) showing asymmetric photocurrent generation under zero bias due to intrinsic photogalvanic effects [62]. To improve spectral reach, several studies have employed metamaterials and waveguide integrations. Graphene/BP metasurface detectors developed by Ogawa et al. utilized localized surface plasmon resonance to modulate IR absorption characteristics [63], while Wang and co-workers demonstrated that the BP integrated with lithium niobate photonic waveguides showed 100 ns responses in the telecom range [64]. These hybrid devices highlight the compatibility of BP with photonic circuitry and IR fiber optic communication platforms. Mid-infrared operability and high-temperature resilience are also emerging themes. Yang et al. designed BP/PtSe_2_ Schottky devices capable of maintaining high responsivity (≈25 A/W) up to 470 K, demonstrating BP’s viability in harsh environments [65]. BP’s performance in MWIR surpasses that of commercial III–V photodetectors in room temperature conditions, as reviewed by Higashitarumizu et al. [66]. Meanwhile, Zhu et al. emphasized the importance of BP’s layer dependent tunability and multi-type architecture versatility in their extensive review [67]. Also, Figure 4a,c show the different architectures of device fabrication.

Collectively, these works highlight that black phosphorus, with its unique band structure and anisotropy, stands at the forefront of 2D infrared optoelectronics. Yet challenges remain, including intrinsic air sensitivity, reproducibility in large-scale synthesis, and integration with CMOS-compatible platforms. The field is now rapidly moving toward hybrid and multifunctional device architectures where BP serves as the active layer alongside plasmonic, ferroelectric, and metamaterial components.

### 3.4. MXenes Photodetectors

Two-dimensional (2D) MXenes have emerged as highly promising materials for photodetector applications, owing to their unique electronic and optical properties. As members of the transition metal carbides and nitrides family, MXenes exhibit high electrical conductivity, tunable work functions, and excellent optoelectronic characteristics [70]. These features have driven extensive research into integrating MXenes with various semiconductors and nanostructures, aiming to enhance the performance of photodetectors. This overview highlights recent advancements in 2D MXene photodetectors, focusing on material innovations, device architectures, and key performance metrics.

A major focus in MXene-based photodetectors has been the development of heterojunctions and composite structures to improve charge separation and light absorption. For example, the integration of MXene with bismuth vanadate (BiVO_4_) forms a Schottky junction that significantly enhances photocurrent density and responsivity under visible light illumination [70]. Similarly, Ding et al. demonstrated that van der Waals MXene/gallium nitride (GaN) heterojunctions have high responsivity and operational stability in ultraviolet detection [71]. Rhyu et al. synthesized composite materials, such as MXene decorated with indium oxide (In_2_O_3_) nanoparticles, through sonochemical methods to achieve broadband responsivity spanning ultraviolet, visible, and near-infrared wavelengths. These composites enable flexible photodetectors that maintain high responsivity and mechanical robustness under bending or stretching [72]. High-entropy (HE) MXenes, such as TiVCrMoC_3_$T_{x}$, represent another material innovation, exhibiting superior nonlinear optical properties and ultrafast photoresponse compared with conventional MXenes [73]. Gao and co-workers demonstrated that their stronger saturable absorption makes them suitable for applications in mode-locked lasers and ultrafast photodetection systems. Additionally, surface modifications and functionalization strategies have been employed to tailor MXene properties [73]. For instance, phenylsulfonic acid functionalization can open the bandgap of MXenes, allowing near-infrared photodetection and enabling the fabrication of flexible photodetectors with high responsivity and detectivity [74].

MXene-based photodetectors have demonstrated impressive performance metrics, including high responsivity and detectivity across different wavelength ranges. Zhang et al. reported that MXene/germanium (Ge) Schottky heterostructures exhibited high responsivity as high as 665 mA/W and detectivity of $1.24\times 10^{13}$ Jones at 1550 nm, making them suitable for telecommunications applications [75]. Ghosh and Giri found that MXene/bismuth sulfide (Bi_2_S_3_) composites exhibited peak responsivities of 36.7 A/W in the UV range and 26.2 A/W in the near-infrared range, demonstrating broadband detection capabilities [76]. Li et al. explored that MXene/aluminum gallium nitride (AlGaN) van der Waals junctions achieve a responsivity of 101.85 mA/W under deep ultraviolet illumination, which shows their applicability in UV photodetection [77]. Fast response times and device stability are crucial for practical applications. Ding et al. reported that the rise and decay time of MXene/GaN photodetectors were 8 ms and 14 ms, respectively [71], while MXene/Ge heterostructures exhibit ultrafast response speeds of 1.4 μs (rise) and 4.1 μs (decay) [78]. Stability tests conducted by Zheng and co-workers revealed that MXene/ZnO nanorod photodetectors retain their electrical and optical properties even after 10,000 bending cycles, highlighting their potential for flexible and wearable devices [79]. Broadband and polarization-sensitive detection are additional key features of MXene photodetectors. For example, MXene/bismuth selenide (Bi_2_Se_3_) heterostructures constructed by Nandi et al. detected light throughout a broad spectral range of 300–2000 nm with a responsivity of 7.56 A/W at 980 nm [80]. Furthermore, Nb_2_CT_*x*_-based photodetectors have demonstrated polarization sensitivity with an extinction ratio of 7.6, making them promising candidates for polarization sensitive applications such as optical communication and imaging [81].

**Figure 5:**
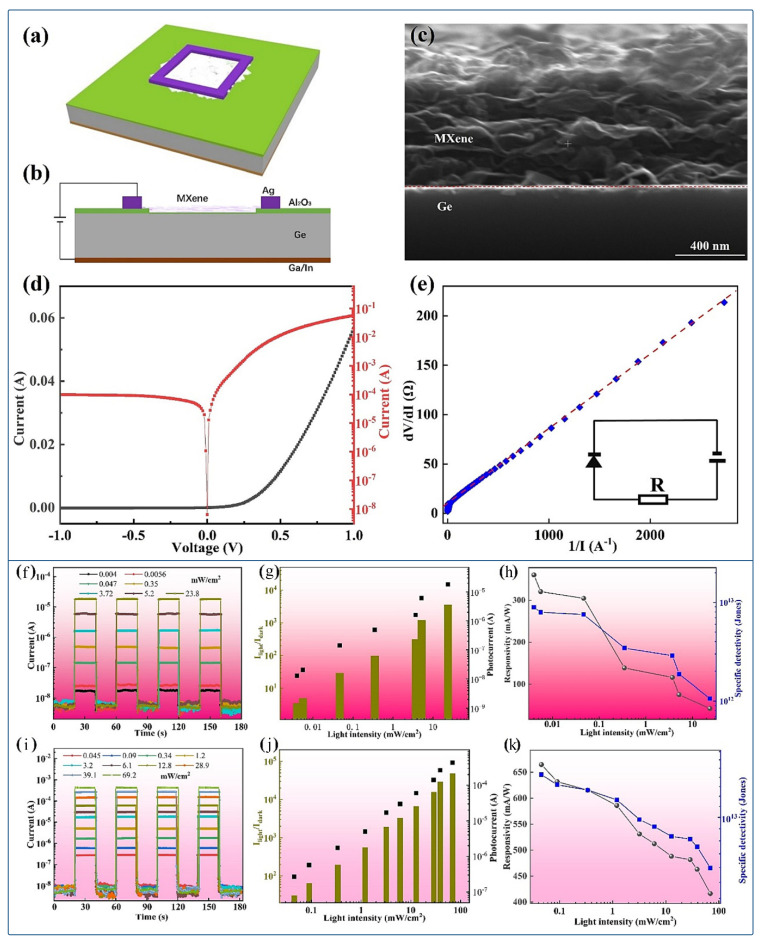
(**a**) The schematic diagram and (**b**) The cross section view of the prepared Ti_3_C_2_T_*x*_/Al_2_O_3_/Ge vdW Schottky junction. (**c**) The cross section SEM image of the vdW Schottky junction. (**d**) The linear (Black line) and semi-logarithmic (Red line) current-voltage curves of the vdW Schottky junction. (**e**) The curve of dV/dI versus 1/I of the vdW Schottky junction, the inset shows the model of equivalent circuit, which consists of an ideal diode and a resistor [75]. (**f**,**i**) The time dependent photoresponse in self-powered mode according to different light intensities at 980 and 1550 nm, respectively. (**g**,**j**) The I_*light*_/I_*dark*_ ratios and photocurrents based on the varying intensities of the incident light at 980 and 1550 nm, respectively. (**h**,**k**) The calculated values of Responsivity (Green line) and Specific detectivity (Blue line) according to the varying light intensities at 980 nm and 1550 nm, respectively [75].

Despite their promising features, MXene photodetectors face several challenges. The intrinsic metallic conductivity of MXenes can limit their use in photodetectors, which often require semiconducting behavior for efficient photoresponse [82]. Scalability and manufacturing remain difficult due to the need for precise control over material quality and interface engineering during large-scale synthesis and device fabrication [82]. Additionally, interfacial defects in heterojunctions can hinder charge transport and increase recombination, reducing device efficiency [75].The detailed results of the experiment conducted by Zhang and co-workers are shown in Figure 5a–k [75]. However, MXenes also present significant opportunities. Their tunable work function allows optimization of heterojunctions for specific photodetection applications [83]. Their inherent mechanical flexibility makes them ideal candidates for flexible and stretchable optoelectronic devices, expanding the potential for wearable technologies [74,84]. Furthermore, emerging materials such as high-entropy MXenes and functionalized MXene derivatives open new avenues for improving photodetection performance and expanding the functional range of these devices [73,74]. Overall, 2D MXenes represent a versatile and powerful class of materials for next-generation photodetectors, with ongoing research continuing to address current challenges and unlock their full potential.

### 3.5. Carbide Photodetectors

**Figure 6:**
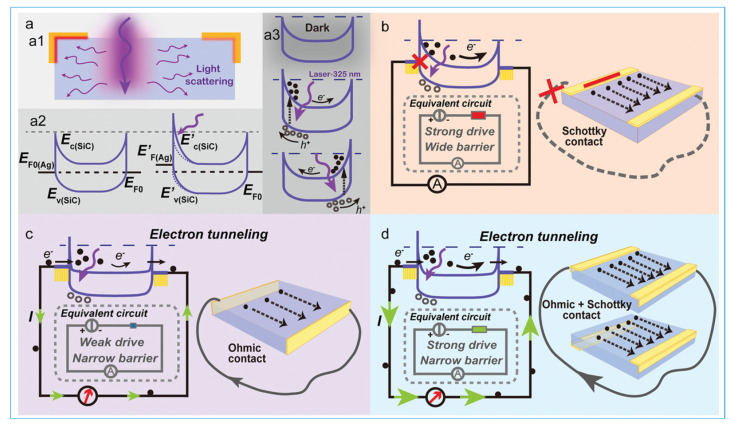
Working mechanism of the self−powered 4H−SiC PD. (**a**) Energy band variation of “Schottky + Ohmic” contact under different illumination conditions. (**a1**) Diagram of laser scattering in SiC crystal; (**a2**) schematic diagram of energy band change after illumination; (**a3**) schematic diagram of energy band changes corresponding to illumination at different positions. (**b**−**d**) The energy band changes of the self−powered 4H−SiC PD and equivalent schematics under Schottky contact (**b**), Ohmic contact (**c**), and “Schottky + Ohmic” contact (**d**), respectively [85].

Carbide-based photodetectors have gained significant traction in optoelectronics due to their exceptional material properties, including wide bandgap, thermal stability, and chemical inertness [85]. Among these, silicon carbide (SiC) is one of the most widely studied materials for ultraviolet (UV) detection [2]. Zhang et al. demonstrated that N-doped 4H-SiC photodetectors exhibit enhanced UV response due to a pyrophototronic effect that amplifies the photocurrent under transient conditions [85]. Also, the working mechanism of N-doped 4H-SiC photodetector is shown in Figure 6a–d [85]. Ali et al. explored multiphoton photocurrent generation in SiC and found that even though the photon energy was below the bandgap, four-photon absorption dominated the photocurrent at 1030 nm femtosecond excitation [86]. In another study, Koller et al. investigated strain-enabled control of vanadium spin qudits in SiC, suggesting the material’s potential for quantum photonics and spin-based sensing applications [87]. Researchers have also focused on heterostructures involving transition metal carbides and SiC to enhance photodetector performance. Vanadium carbide (V_2_C), an MXene, was employed by Altaleb et al. to construct a mid-infrared (mid-IR) photodetector, achieving a responsivity of 2.65 A/W at 2μm and demonstrating compatibility with on-chip photonic circuits [88]. From a theoretical standpoint, Roy et al. performed first-principles simulations of quantum dots (QDs) composed of SiC and GeC, showing spin-split energy states, SWIR emission, and enhanced gas sensing due to strong charge localization at heterojunctions [89]. Such theoretical insights were complemented by experimental studies. For example, Remeš et al. fabricated thin hydrogenated amorphous SiC films embedded with Ge nanocrystals and observed increased optical absorption in the near-infrared region, though with some degradation after high-temperature annealing [90].

Hybrid structures combining SiC with borides and organic materials have also been developed to expand photodetection capabilities. These hybrid approaches address the limitations of individual materials by synergizing their optical and electronic properties. Graphene and other 2D materials have also been integrated with carbides to exploit their tunable electronic properties. Yang et al. engineered an all-optical modulator based on a CdS/Graphene/Ge sandwich, demonstrating responsivity tunability from −3376–+3584 A/W, making it suitable for neuromorphic computing [91]. Additionally, Zhang et al. and Du et al. reviewed photodetectors based on graphitic carbon nitride and highlighted its advantages in flexible, broadband, and cost-effective detection platforms [92,93]. A variety of fabrication methods and device architectures have been employed to further improve performance. Lionas et al. compared solution-processed and chemical vapor-deposited multi-wall carbon nanotube devices on Si_3_N_4_/n-Si, finding that UV sensitivity was superior in solution-processed devices [94]. Cai et al. used femtosecond laser pulses to probe nonlinear photocurrent effects in SiC, confirming the material’s robustness and suitability for high-power optical applications [86]. Zhang et al. showed that pyro-phototronic SiC photodetectors could perform imaging and signal modulation at room temperature [85]. Meanwhile, Woo et al. reported high-detectivity dual-band infrared photodetectors based on dislocation-assisted gain mechanisms using heteroepitaxial layers involving carbide interfaces, expanding the utility of such devices to mid-IR applications [95]. In summary, recent advances underscore the versatility of carbide-based photodetectors. Their performance can be significantly enhanced through heterostructure engineering, organic and boride integration, and quantum-level modifications. These devices offer high responsivity across UV to mid-IR ranges, improved detectivity, thermal stability, and potential integration into flexible and neuromorphic platforms. Collectively, these findings position carbide photodetectors as leading candidates for next-generation sensing technologies in harsh and multifunctional environments.

### 3.6. Chalcogenide Photodetectors

Chalcogenide materials, composed of elements from group 16 (chalcogens) combined with metals or metalloids, have garnered significant attention for next-generation optoelectronic applications due to their tunable bandgaps, high optical absorption coefficients, and compatibility with flexible substrates. In particular, layered chalcogenide compounds such as bismuth, antimony, and tin-based materials offer compelling advantages for photodetection across the visible-to-near-infrared (NIR) spectrum. Their unique electronic structures, coupled with solution-processable or vapor-phase synthesis routes, enable the fabrication of high-performance, often flexible, photodetectors [96,97,98,99,100]. This section explores the recent progress in chalcogenide-based photodetectors, highlighting their operating principles, material characteristics, and key performance metrics demonstrated in practical device configurations.

In the visible to near-infrared (NIR) wavelength range, bismuth chalcogenide nanomaterials have emerged as promising candidates for photodetection applications due to their unique properties. Bismuth telluride (Bi_2_Te_3_) nanoplates grown by controlled chemical vapor deposition (CVD) have shown excellent photoresponse for visible light detection, exhibiting a high responsivity of 55.06 A/W and specific detectivity of $5.9\times 10^{7}$ Jones at 850 nm [96]. Wang et al. reported that these devices demonstrated stable photoswitching behavior and maintained good performance after mechanical bending, indicating potential applications in flexible optoelectronics. Similarly, Liu et al. developed ultrafast and high-flexibility NIR photodetectors based on Bi_2_Se_3_ nanobelts grown via catalyst-free van der Waals epitaxy [97]. These devices achieved a rapid photoresponse (response time of 37 μs and decay time of 62 μs) with a responsivity of 10.1 mA/W and detectivity of $4.63\times 10^{8}$ Jones at 735 nm. More recently, Wang et al. fabricated broadband photodetectors based on Bi_2_O_2_Se nanoplates grown using chemical vapor deposition [98]. These square-shaped Bi_2_O_2_Se nanoplates showed exceptional performance at 77 K with a high responsivity of 523 A/W, specific detectivity of $1.37\times 10^{11}$ Jones, and external quantum efficiency of 162,119.44% under 400 nm illumination. Furthermore, the Bi_2_O_2_Se nanoplates enabled high-quality full-color imaging in the visible spectrum, highlighting their potential for advanced optoelectronic applications.

**Figure 7:**
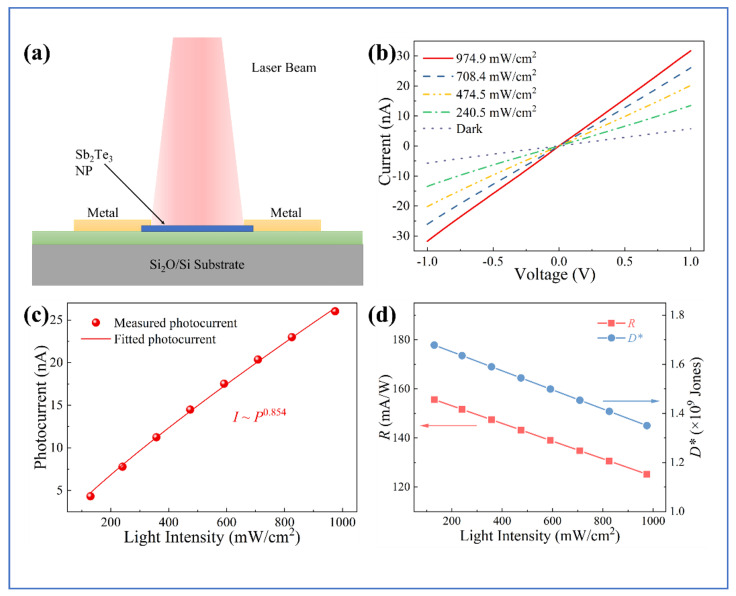
(**a**) Schematic illustration of the photodetection setup, where a laser beam irradiates the Sb_2_Te_3_ nanoparticle (NP)−based photodetector fabricated on a SiO_2_/Si substrate with metal electrodes. (**b**) Curren–voltage (I–V) characteristics of the device under 850 nm light illumination at various optical power densities (974.9, 708.4, 474.5, and 240.5 mW/cm^2^), along with the dark condition, measured at room temperature with a 1 V bias. (**c**) Photocurrent as a function of incident light intensity, showing a power-law dependence, indicating the nonlinear photoresponse behavior. (**d**) Responsivity (R) and specific detectivity (D^*^) of the device as a function of light intensity, both showing a decreasing trend with increasing power density. All measurements were performed at room temperature under a constant bias of 1 V [100].

Similar to bismuth chalcogenides, antimony telluride (Sb_2_Te_3_) possesses a rhombohedral crystal structure with quintuple layers stacked by van der Waals interactions [99]. This structure, combined with its narrow bandgap (0.15–0.3 eV) and high carrier mobility (200 cm^2^$\;V^{-1}s^{-1}$) [101,102], makes Sb_2_Te_3_ nanoplates promising for photodetection applications. Zhang et al. recently demonstrated high-performance photodetectors based on CVD-grown Sb_2_Te_3_ nanoplates [100]. Figure 7a illustrates the photodetection measurement setup used to characterize their Sb_2_Te_3_ nanoplate devices. The I–V characteristic curves (Figure 7b) exhibit symmetric and linear behavior under different illumination intensities, indicating Ohmic-like contacts between the Sb_2_Te_3_ nanoplates and metal electrodes. The photocurrent follows a power law relationship with light intensity ($I_{ph}\approx P^{0.854}$), as shown in Figure 7c, confirming that the photoconductive mechanism dominates the device operation. Figure 7d presents the light intensity-dependent responsivity and specific detectivity of the Sb_2_Te_3_ nanoplate photodetector. Under 850 nm illumination with a light power intensity of 130.0 $\mathrm{mW}\;{\mathrm{cm}}^{-2}$, the devices achieved a responsivity of 155.6 $\mathrm{mA}\;W^{-1}$ and a specific detectivity of $1.68\times 10^{9}$ Jones. Both parameters decrease with increasing light intensity due to enhanced carrier trapping and recombination at higher carrier concentrations. These photodetectors demonstrated broadband spectral response from 400 to 980 nm with maximum sensitivity at 850 nm. Remarkably, the devices exhibited ultrafast photoresponse characteristics with rise and decay times both measuring just 64 μs, significantly faster than many other chalcogenide-based photodetectors. The Sb_2_Te_3_ nanoplate photodetectors also maintained stable performance over 180 photoswitching cycles, confirming their excellent potential for practical NIR photodetection applications.

Expanding beyond Bi- and Sb-based systems, tin chalcogenides such as tin sulfide (SnS) and tin selenide (SnSe) also offer promising attributes for photodetector design. These materials crystallize in an orthorhombic-layered structure analogous to black phosphorus and exhibiting high optical absorption coefficients along with intrinsic p-type conductivity originating from native Sn vacancies [103,104]. Photodetectors based on SnSe nanoplates have demonstrated excellent room temperature performance, with a responsivity of 1.32 A/W and a high photoswitching ratio of 176.14 under 400 nm laser illumination at a bias voltage of 1 V [105]. Further enhancing the scope of chalcogenide photodetection, tin telluride (SnTe), a representative topological crystalline insulator with a rock-salt structure, possesses a narrow bandgap and multiple topologically protected metallic surface states that significantly enhance light–matter interactions and facilitate efficient photocarrier separation, particularly in the near-infrared region [106,107]. Photodetectors based on ultrathin SnTe nanoplates exhibit outstanding NIR performance, achieving a responsivity of 698 mA/W under 980 nm illumination at room temperature without gate modulation [108].

In summary, chalcogenide nanomaterials have shown immense promise for broadband photodetection, tunable electronic properties, and compatibility with scalable fabrication methods. From the high responsivity and flexibility of Bi_2_Te_3_ and Bi_2_Se_3_ devices to the ultrafast response and stability of Sb_2_Te_3_ nanoplates, each chalcogenide system offers distinct advantages suited for specific application domains. Additionally, tin-based compounds such as SnSe and SnTe have expanded the detection capabilities into both the visible and NIR regions, leveraging their structural anisotropy and topologically enhanced surface states. Collectively, these advances reinforce the potential of chalcogenide materials as foundational building blocks for future flexible, high-performance, and broadband photodetectors.

## 4. Performance Metrics of Different 2D-Based Photodetectors

Two-dimensional (2D) materials have emerged as promising platforms for next-generation photodetectors due to their atomically thin structures, diverse bandgaps, and tunable electronic and optical properties. These characteristics enable device miniaturization, mechanical flexibility, and spectral versatility spanning from ultraviolet (UV) to far-infrared (FIR) regimes. Evaluating the photodetection performance of 2D materials typically involves metrics such as responsivity (R), detectivity (D*), response time, noise-equivalent power (NEP), and spectral range [17]. These parameters serve as a basis for benchmarking different materials and understanding their suitability for specific applications such as imaging, sensing, optical communication, or environmental monitoring.

Graphene, for instance, offers ultrafast response times and broadband absorption but suffers from low responsivity due to its gapless nature [109]. However, device engineering strategies such as photogating, plasmonic enhancement, and hybridization with other semiconductors have significantly boosted its responsivity and detectivity across a wide spectral range. Transition metal dichalcogenides (TMDCs) such as MoS_2_ and WSe_2_ exhibit high responsivity and detectivity in the visible spectrum owing to their direct bandgaps and strong light–matter interactions [39,110]. Despite slower response times compared with graphene, TMDC-based photodetectors benefit from large on/off ratios and reduced dark currents, making them well suited for visible light imaging and sensing applications. Black phosphorus (BP) combines moderate responsivity with fast response times and is particularly suitable for mid-infrared detection due to its tunable direct bandgap ranging from 0.3 eV to 2 eV [111]. BP devices exhibit high polarization sensitivity and an anisotropic photoresponse, which offer a unique advantage in polarization-resolved imaging and sensing applications. However, BP’s environmental instability remains a key challenge, often requiring encapsulation or passivation strategies to maintain long-term performance.

2D carbides, such as molybdenum and tungsten carbides, have shown promise due to their metallic conductivity and thermal stability. Though less extensively studied, they demonstrate UV–visible photoresponse mechanisms linked to surface plasmon resonance and defect-induced absorption. Still, their relatively low responsivity and detectivity necessitate improved material quality and interface engineering. MXenes, a family of 2D transition metal carbides and nitrides, offer broadband operation, fast response (typically in the microsecond regime), and high environmental resilience. Functional surface terminations and high carrier mobility enhance their photodetection properties, although issues such as high dark current and limited spectral selectivity remain challenges. Meanwhile, 2D chalcogenides like Bi_2_Se_3_, Sb_2_Te_3_, and SnSe extend the operational wavelength to the mid-IR and FIR regions. These materials feature narrow bandgaps, high thermoelectric coefficients, and topological surface states that can be exploited for novel detection mechanisms, though their responsivity is typically lower than TMDCs and BP. Finally, heterostructures and hybrid systems combining different 2D materials or integrating them with quantum dots and plasmonic layers have demonstrated enhanced photodetection performance by leveraging synergistic effects such as interfacial charge transfer and photogating [112,113].

To provide a comprehensive comparison, we summarize the key performance metrics—including responsivity, detectivity, response time, and spectral range—for graphene, TMDCs, BP, carbides, MXenes, chalcogenides, and their hybrid systems in Table 2. This comparison highlights the quantitative comparison of the 2D materials discussed in this review.

## 5. Applications of 2D Photodetectors

Two-dimensional (2D) photodetectors have emerged as promising candidates for a wide range of optoelectronic applications due to their exceptional responsivity, ultrafast response times, mechanical flexibility, and broad spectral tunability. Their atomic thinness and van der Waals structures facilitate integration into both rigid and flexible substrates, enabling new device architectures in diverse technological fields.

In high-speed optical communication, 2D materials such as graphene and transition metal dichalcogenides (TMDCs) offer superior carrier mobility and ultrafast photocarrier dynamics. These properties have enabled the demonstration of photodetectors with bandwidths exceeding 100 GHz, suitable for next-generation data communication systems [119]. The compatibility of 2D materials with complementary metal oxide semiconductor (CMOS) platforms and silicon photonics further facilitates their integration into miniaturized on-chip photonic circuits and optical interconnects.

Beyond telecommunications, 2D photodetectors have extensive applications in broadband imaging and sensing. For example, Bi_2_O_2_Se nanoplate-based devices demonstrate high responsivity (523 A/W) and detectivity ($1.37\times 10^{11}$ Jones) at 77 K under 400 nm illumination, allowing full color imaging in the visible range [98]. Similarly, Sb_2_Te_3_ nanoplate photodetectors offer broadband spectral response (400–980 nm) and ultrafast response times of 64 μs, highlighting their potential for high-speed visible and NIR imaging [100].

In biomedical sensing and diagnostics, 2D materials such as black phosphorus (BP) and SnSe exhibit strong NIR absorption and biocompatibility, enabling non-invasive imaging in spectral windows where tissue transparency is optimal [105]. Their inherent flexibility and high surface-to-volume ratio also enhance sensitivity to biomolecules and environmental analytes, supporting applications in wearable biosensors and environmental monitors. Gleichzeitig, narrow-bandgap chalcogenides such ass SnTe and Bi_2_Se_3_, which exhibit strong NIR absorption and topologically protected surface states, areactively being explored for room temperature mid-infrared (MIR) and long-wave infrared (LWIR) detection. These capabilities are particularly valuable for thermal imaging, night vision, industrial process monitoring, and aerospace surveillance [97,108].

Finally, the mechanical flexibility and transparency of 2D materials make them ideal for next-generation human–machine interfaces, flexible displays, and conformal electronics integrated on soft or curved surfaces. The convergence of high performance, integrability, and mechanical adaptability positions 2D photodetectors at the forefront of flexible and wearable optoelectronics.

## 6. Challenges and Future Perspectives

The widespread deployment of 2D material-based photodetectors faces a range of challenges that are material-specific but often interconnected. Addressing these limitations while exploring the potential of emerging heterostructures and hybrid devices will be crucial to unlocking their full commercial potential. Below, we provide tailored future directions for each material class discussed in this review.

1. Graphene’s exceptional carrier mobility and broadband absorption make it a promising candidate for photodetectors. However, its zero bandgap leads to low responsivity and high dark currents. Enhancing light–matter interactions through integration with plasmonic structures, optical cavities, or hybridization with semiconductors like perovskites can mitigate these issues [120]. For instance, integrating graphene with perovskite materials has shown significant improvements in photodetector performance. Additionally, developing large-area, uniform chemical vapor deposition (CVD) growth and transfer-free integration techniques is crucial for scalability and compatibility with complementary metal oxide semiconductor (CMOS) technology [120].
2. TMDCs, such as MoS_2_ and WSe_2_, offer direct bandgaps and strong light–matter interactions, making them suitable for photodetection applications. Challenges include achieving wafer-scale synthesis with minimal defects and integrating these materials into existing electronic platforms [121]. Future research should focus on strain and phase engineering to tailor electronic properties and on developing heterostructures with other 2D materials to enhance performance. Recent studies have highlighted the potential of TMDCs in self-powered photodetectors, emphasizing the need for innovative approaches to overcome current limitations [121].
3. BP exhibits a thickness-dependent direct bandgap, making it ideal for mid-infrared photodetection. However, its instability in ambient conditions poses significant challenges. Encapsulation strategies using materials like hexagonal boron nitride (h-BN) or aluminum oxide (Al_2_O_3_) are essential to enhance stability [66]. Moreover, integrating BP with photonic structures can lead to polarization-sensitive and broadband photodetectors. Advancements in understanding BP’s photophysics are paving the way for its application in mid-infrared optoelectronics.
4. MXenes, a family of 2D transition metal carbides and nitrides, exhibit high conductivity and tunable surface chemistry, making them attractive for photodetector applications. The primary challenge lies in their metallic nature, which can limit photoresponse [122]. Surface functionalization and hybridization with semiconducting materials can induce semiconducting behavior, enhancing photodetection capabilities. Recent reviews have discussed materials engineering strategies to optimize MXene-based photodetectors [122].
5. Carbide materials, such as silicon carbide (SiC), are known for their wide bandgaps and thermal stability, making them suitable for ultraviolet and high-temperature photodetection. Challenges include controlling doping levels and improving interface quality to enhance device performance. Future research should explore hybrid structures combining carbides with other 2D materials to leverage their complementary properties [123].
6. Chalcogenide materials, including bismuth telluride (Bi_2_Te_3_) and tin telluride (SnTe), offer high optical absorption and are promising for infrared photodetection. However, issues related to material stability and integration need to be addressed. Developing solution-processable fabrication methods and exploring heterostructures with other 2D materials can enhance device performance [124].

## 7. Conclusions

This review has provided a comprehensive summary of recent advances in two-dimensional (2D) material-based photodetectors, encompassing a wide range of materials including graphene, transition metal dichalcogenides (TMDCs), black phosphorus (BP), MXenes, carbides, and chalcogenides. Each material class exhibits unique advantages: graphene offers ultrafast and broadband detection; TMDCs provide high responsivity in the visible spectrum; BP supports mid-infrared detection with anisotropic properties; MXenes and carbides show promise for flexible and UV-resilient systems; and chalcogenides enable broadband and topologically enhanced detection. Across these platforms, various detection mechanisms such as photovoltaic, photogating, photoconductive, and plasmonic enhanced effects are exploited for tailored photodetector performance. Despite the rapid progress, several key challenges remain. These include poor environmental stability (especially in BP), limited scalability and uniformity of large-area synthesis (notably in TMDCs and MXenes), low quantum efficiency in pristine graphene, and difficulty integrating 2D materials with existing CMOS and photonic platforms. These challenges hinder the commercialization and broader deployment of high-performance 2D photodetectors. Looking ahead, the community should prioritize research in several strategic directions. These include wafer-scale synthesis of high-quality 2D materials, robust encapsulation strategies for unstable materials, and seamless integration with silicon photonics and flexible substrates. Promising solutions also lie in emerging hybrid systems such as perovskite 2D, quantum dot 2D, or 2D ferroelectric heterostructures, which can offer synergistic advantages in sensitivity, tunability, and multifunctionality. Moreover, open questions remain regarding device stability under ambient conditions, long-term reliability, scalable manufacturing, and the development of unified benchmarking metrics across platforms. To realize the full potential of 2D photodetectors in next-generation applications such as integrated optical communication, wearable sensors, biomedical imaging, and neuromorphic sensing, a coordinated interdisciplinary effort is required. This includes not only materials innovation but also progress in device architecture, system-level integration, and industrial-scale fabrication.

## Figures and Tables

**Table 1:** Comparison of photodetection mechanisms in 2D materials.

Mechanism	Driving Principle	Required Bias?	Key Advantage	Limitation	Typical Application
Photovoltaic Effect	Internal electric field	No	Low power, fast response	Limited to built-in field zones	PN junction devices
Photothermoelectric Effect	Temperature gradient (Seebeck)	No	Thermopower-based detection	Requires asymmetry, slower	THz/MIR sensors
Photoconductive Mechanism	Light-induced carrier generation	Yes	High gain, tunable	Requires bias, more noise	Visible/NIR photodetectors
Bolometric Effect	Resistance change due to heating	Yes	Sensitive in IR	Slow response	IR thermal detectors
Carrier Trapping/Photogating	Long-lived trapped carriers	Yes	Very high responsivity	Slow switching speed	Low-light imaging
Heterostructures Effect	Built-in fields at interfaces	Depends	Efficient separation, tunability	Complex fabrication	Broadband sensors
Plasmon-Enhanced Effect	Local field enhancement	Depends	Boosted absorption/sensitivity	Complexity, limited bandwidth	Ultra-sensitive detectors

**Table 2:** Performance metrics of selected 2D-based photodetectors.

Material	Spectral Range	Responsivity (A/W)	Detectivity (Jones)	Response Time (ms)	Reference
Graphene on SOS slot waveguide	MWIR (3–5 μm)	0.21–0.26	−	−	[9]
Graphene with ChG waveguide	2.0–2.55 μm	0.25	−	0.0023	[33]
MoS_2_	Visible (660 nm)	880	$10^{13}$	50	[39]
Te_2_-regulated black AsP	2–8 μm	0.1–10	$10^{9}–10^{11}$	–	[53]
BP and Reduced GO	532–2200 nm	12	$2.4\times 10^{9}$	–	[56]
BP with asymmetric AU	405–1064 nm	34	$2.47\times 10^{11}$	–	[57]
BP with asymmetric AU	2200 nm	20	$1.94\times 10^{11}$	–	[57]
BP and MoS_2_	3–5 μm	–	$10^{10}$	–	[60]
BP in nanowire	–	802.42	–	$6.36\times 10^{-7}$	[61]
Monolayer BP-Bi	Visible–NIR	0.133 (ferroelectric direction)	–	–	[62]
Monolayer BP-Bi	Visible–NIR	0.0047 (Zigzag direction)	–	–	[62]
BP with lithium niobate	1550 nm	0.148	–	–	[64]
BP and PtSe_2_	532–2200 nm	25 at 1850 nm	$6.4\times 10^{11}$ at 1850 nm	–	[65]
p-type BP and n-type MoTe_2_	1500–1630 nm	0.577 (intrinsic), 0.709 (electrostatically tuned)	–	$1\times 10^{-6}$	[68]
BP	3.7–7.7 μm	0.518–0.0022	–	–	[69]
2D BiVO_4_/MXene (Ti_3_C_2_T_*x*_)	447 nm	0.79	–	8 (rise) and 14 (fall)	[70]
MXene/GaN	UV	0.6816 at 360 nm	$7.65\times 10^{13}$	–	[71]
In_2_O_3_-decorated MXene nanosheets	UV–NIR	121.6	$1.064\times 10^{12}$	–	[72]
Phenylsulfonic acid groups modified MXene	1064 nm	850	$3.69\times 10^{11}$	–	[74]
MXene/Ge with Al_2_O_3_ interfacial layer	–	0.665	$1.24\times 10^{13}$	0.0735 (rise) and 0.0815 (fall)	[75]
MXene/Bi_2_S_3_ nanorod composite	300–1550 nm	36.7 at 300 nm and 26.2 at 780 nm	–	0.3	[76]
Nb_2_CT_*x*_ MXene/AlGaN	254 nm	0.101	–	21 (rise) and 22 (fall)	[77]
MXene/n-type Ge	UV–NIR	3.14	$2.14\times 10^{11}$	0.0014 (rise) and 0.0041 (fall)	[78]
MXene nanosheets/ZnO nanorods	368 nm	0.1422	$2.03\times 10^{10}$	12200 (rise) and 3900 (fall)	[79]
MXene/CVD-grown Bi_2_Se_3_	300–1550 nm	6.96 at 808 nm and 7.56 at 980 nm	$6.31\times 10^{12}$ at 808 nm and $6.85\times 10^{12}$ at 980 nm	0.0197 (rise) and 0.0352 (fall)	[80]
Nb_2_CT_*x*_ and Nd_2_CT _*X*_ at MoS_2_	Vis–NIR	$8.4\times 10^{-4}$	–	–	[81]
MXene/InGaN	Visible	0.133	$2.81\times 10^{11}$	0.03749 (rise) and 0.11 (fall)	[83]
N-doped 4H–SiC	UV	$9.12\times 10^{-6}$	–	270	[85]
TaC:Cu alloy on 4H-SiC substrate	405 nm	1.66	$2.69\times 10^{8}$	–	[2]
MoS_2_/W$O_{3-x}$-MoS_2_	Visible–NIR	$1.8\times 10^{4}$	$4.62\times 10^{13}$	0.24	[114]
InSe/WSe_2_ vdW	532–1100 nm	$8.30\times 10^{2}$	$2.81\times 10^{14}$	0.010	[115]
WS_2_/ZnO-QD	UV–visible	$1.12\times 10^{3}$	$3.23\times 10^{12}$	$1.5\times 10^{-6}$	[116]
MoSe_2_/AIGS-QDs	Visible–NIR	14.3	$6.4\times 10^{11}$	−	[117]
MoTe_2_/Ta_2_NiSe_5_ dual HJ	400–1550 nm	0.539	$2.3\times 10^{13}$	$3.5/4.2\times 10^{-3}$	[118]
Bi_2_Te_3_ nanoplates	850 nm	55.06	$5.9\times 10^{7}$	−	[96]
Bi_2_Se_3_ nanobelts	735 nm	0.0101	$4.63\times 10^{8}$	0.037	[97]
Bi_2_O_2_Se nanoplates	400 nm	523	$1.37\times 10^{11}$	−	[98]
Sb_2_Te_3_ nanoplates	400–980 nm (max at 850 nm)	0.1556	$1.68\times 10^{9}$	0.064	[100]
SnSe nanoplates	400 nm	1.32	−	−	[105]
SnTe nanoplates	980 nm	0.698	−	−	[108]

## Data Availability

The data and codes that support this work are available from the corresponding author upon reasonable request.

## Abbreviations

List of Acronyms	
2D	Two-dimensional
A/W	Ampere per watt
APTES	Aminopropyltriethoxysilane
APS	Active pixel sensor
BP	Black phosphorus
CMOS	Complementary metaloxide semiconductor
CVD	Chemical vapor deposition
dB	Decibel
DLP	Dember-like photocurrent
ECM	Electrochemical metallization
FET	Field-effect transistor
FIR	Far-infrared
GHz	Gigahertz
HMM	Hyperbolic metamaterial
III–V	Group III–V compound semiconductors
InGaAs	Indium gallium arsenide
IoT	Internet of Things
IR	Infrared
Jones	Unit of specific detectivity
MEMS	Micro-electro-mechanical systems
MeV	Milli electron volt
MHz	Megahertz
MIR	Mid-infrared
MoS_2_	Molybdenum disulfide
MoSe_2_	Molybdenum diselenide
MoTe_2_	Molybdenum ditelluride
MWIR	Mid-wave infrared
MXene	Transition metal carbide/nitride
NEP	Noise equivalent power
NIR	Near-infrared
OTS	Octadecyltrichlorosilane
PD	Photodetector
pJ	Picojoule
PL	Photoluminescence
QE	Quantum efficiency
RGO	Reduced graphene oxide
SAM	Self-assembled monolayer
Si	Silicon
SiN	Silicon nitride
SnS	Tin sulfide
SnSe	Tin selenide
SnTe	Tin telluride
SNR	Signal-to-noise ratio
SWIR	Short-wave infrared
Te	Tellurium
TMDC	Transition metal dichalcogenide
UV	Ultraviolet
VCM	Valence change memory
vdW	van der Waals
WSe_2_	Tungsten diselenide
WS_2_	Tungsten disulfide
